# Glandular odontogenic cyst of the maxilla: a case report and literature review

**DOI:** 10.11604/pamj.2016.25.116.10879

**Published:** 2016-10-26

**Authors:** Nigel Roque Figueiredo, Ajit Dattatray Dinkar, Manisha Maruti Khorate

**Affiliations:** 1 Oral Medicine, Diagnosis and Radiology Department, Goa Dental College & Hospital, Bambolim-Goa, India

**Keywords:** Jaw, maxilla, odontogenic cyst

## Abstract

Glandular Odontogenic Cyst is a relatively rare cyst of odontogenic origin, which shows glandular or salivary features that are thought to indicate the pluripotentiality of odontogenic epithelium. It is seen in middle-aged adults, and commonly involves the anterior region of the jaws, especially the mandible. It shows non-specific clinico-radiographic findings which may resemble other lesions, but has characteristic histopathologic features which help in its diagnosis. This paper reports an unusual presentation of a glandular odontogenic cyst which was diagnosed in a 64-year old female in the posterior maxilla, along with a literature review of this cyst, especially the cases reported in India in the past.

## Introduction

The Glandular Odontogenic Cyst (GOC) is a rare developmental cyst of the jaws. It was initially described by Padayachee and van Wyk in **1**987, who called it ‘Sialo-Odontogenic Cyst’ due to the presence of mucous cells and pools of mucin in the epithelial lining, and the fact that mucous pools are often lined by eosinophilic cuboidal cells which resemble salivary gland ducts [1]. Gardner et al in 1988 suggested the name ‘Glandular Odontogenic Cyst’ because the cyst wall epithelium was odontogenic and contained mucin elements with absence of salivary tissue [2]. In 1992, GOC was included in the WHO typing of tumors under the term GOC or sialo-odontogenic cyst [3]. An important feature of this cyst is that its recognition on the basis of clinical and radiographic features is practically impossible, and only histopathological examination allows for a certain diagnosis [4]. This case report describes a case of glandular odontogenic cyst in a 64-year old female which was diagnosed and treated in our institution.

## Patient and observation

**Figure 1:**
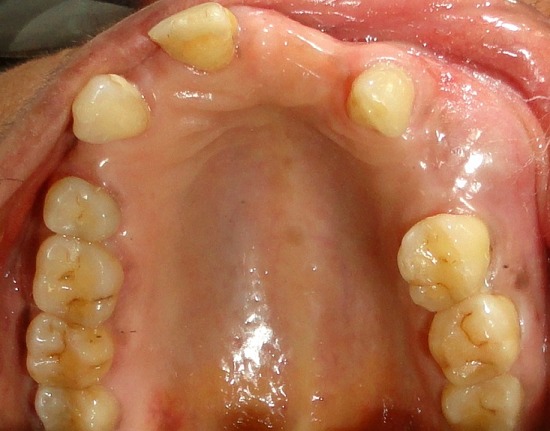
Pre-operative intra-oral view

**Figure 2:**
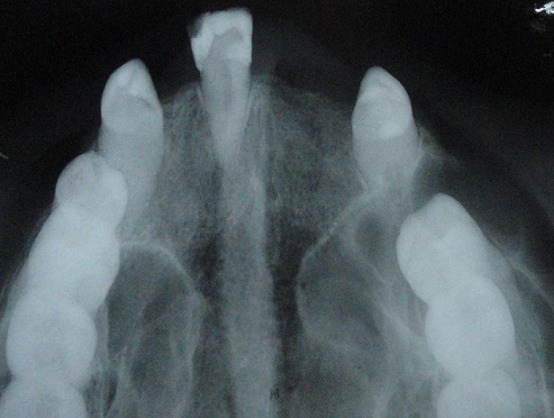
Maxillary true occlusal radiograph showing expansion of buccal cortical plate in region of 23, 24, 25 and 26

**Figure 3:**
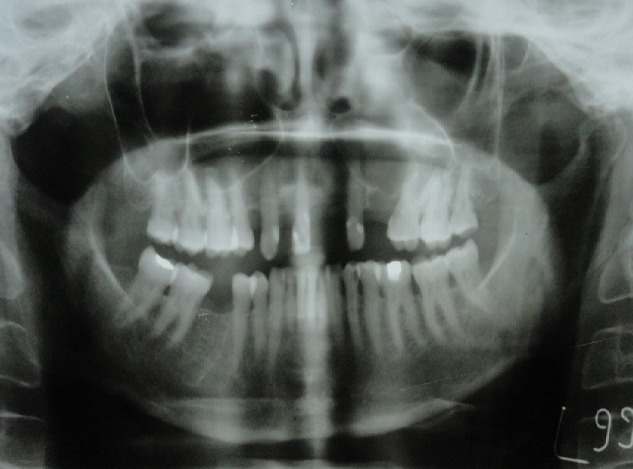
Panoramic radiograph showing an oval-shaped unilocular radiolucency with thin corticated borders extending from 23 to 26 regions

A 64-year-old female patient reported to our out-patient department with a chief complaint of swelling over the left side of the face since two months. History revealed that the swelling was initially small in size and had gradually increased to its present size. On extra-oral examination, a diffuse swelling was seen over the left cheek region, extending from the left nasolabial fold area, posteriorly to the malar region and supero-inferiorly from 2 cms below the infra-orbital margin to 1 cm away from the left angle of mouth, measuring around 3 x 2.5 cms. The overlying skin was normal with no evidence of any discharge. On palpation, the swelling was firm in consistency, tender, non-pulsatile and non-compressible, with no local rise in temperature. Intra-oral examination revealed a diffuse swelling in the left maxilla and buccal vestibule, extending from 23 till the 26 region (Figure 1). The swelling was firm in consistency with a smooth surface and tender on palpation. On examination of the teeth, 23 were restored, while 24 and 25 were missing. No tenderness or mobility of the teeth in the area was noted. An intra-oral periapical radiograph showed a large unilocular radiolucency with a well-defined thin corticated border extending from the distal aspect of 23, upto the 26 region. A maxillary true occlusal view showed a unilocular radiolucent lesion in the 23, 24, 25, 26 region causing expansion of the buccal cortical plate in the affected area (Figure 2). Panoramic radiography showed a well-defined oval-shaped unilocular radiolucency with thin corticated borders extending from the mesial aspect of 23 up to the 26 region, superiorly involving the maxillary sinus and inferiorly the maxillary alveolar crest. The internal structure was completely radiolucent. There was no evidence of root resorption or displacement of 23 or 26 (Figure 3).

**Figure 4:**
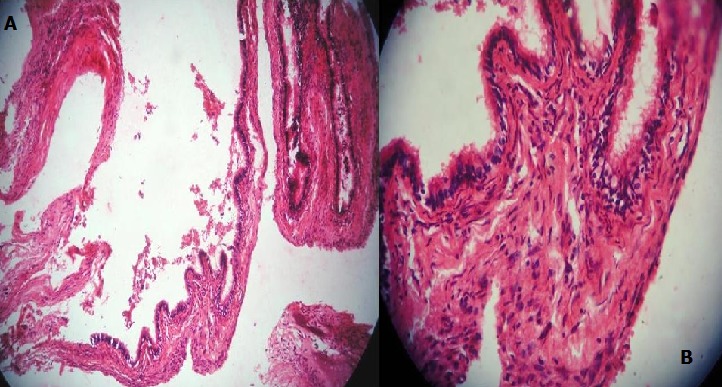
Photomicrograph showing a cystic lesion with papillary projections lined by pseudostratified columnar epithelium with some mucous pools and pseudoglandular areas (a) H&E stain at 10x magnification, (b) H&E stain at 40x magnification

**Figure 5:**
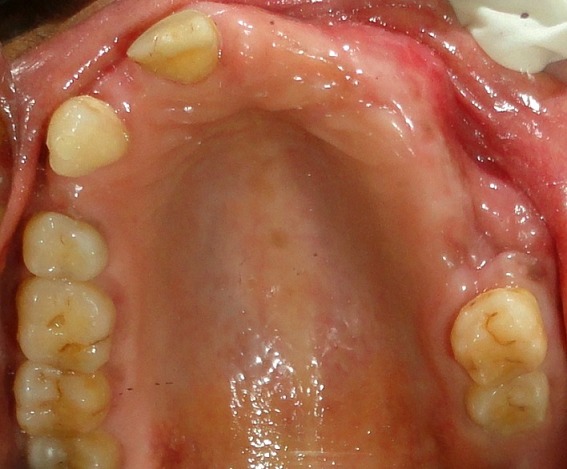
Post-operative intra-oral view

**Figure 6:**
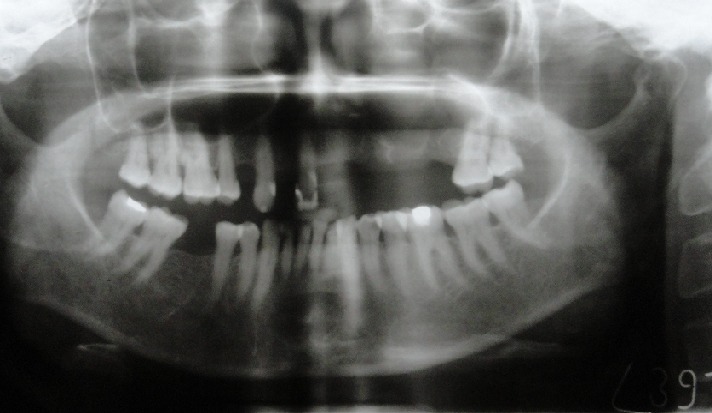
Post-operative panoramic radiograph

Based on the history, clinical and radiographic findings, a provisional diagnosis of a residual cyst was made, with a differential diagnosis of an odontogenic cyst or tumor. The lesion was treated conservatively with careful enucleation and curettage. Histopathological examination showed a cystic lining with a pseudoglandular pattern in some areas, with other areas showing a pseudostratified columnar ciliated epithelium with goblet cells, some mucous pools / crypts (Figure 4). The connective tissue showed presence of collagen fibers, fibroblasts, blood vessels, areas of osteoid formation, and a mild chronic inflammatory cell infiltrate. Thus, based on the histopathology of the enucleated tissue, a final diagnosis of a glandular odontogenic cyst was made. Post-operative healing was uneventful. The patient has been under regular follow-up for the last 2 years, during which time no recurrence has been noted (Figure 5, Figure 6).

## Discussion

GOC is a rare lesion with a frequency rate of only 0.012% to 1.3% of all the jaw cysts [5]. Till date, less than 200 cases of this lesion have been reported in literature. A literature review performed using the search terms “glandular odontogenic cyst”, “sialo-odontogenic cyst” on the PubMed interface yielded only 19 cases of histopathologically confirmed GOC that were reported in India, which have been detailed in Table 1, Table 2. GOC usually occurs in middle-aged adults, most often after the fourth decade of life, with a slight male predilection. It is four times more common in the mandible as compared to the maxilla and has a predilection for the anterior region of the jaws [20]. The present case differs from the literature in that it was diagnosed in a 64-year old female, and occurred in the maxilla extending antero-posteriorly from the canine to the first molar region, which is not commonly seen. Clinically features of this cyst are not specific, with lesions usually presenting as asymptomatic swellings. The radiographic appearance also varies and is not pathognomonic. It may present as a unilocular or multilocular radiolucency, which usually shows well-defined borders [20]. Occasionally, scalloping of the border may be noted, while root resorption and displacement are not commonly seen [3]. Radiographically, GOC may resemble lesions like radicular cyst, keratocystic odontogenic tumor, ameloblastoma and central giant cell granuloma. The radiographic features of our case were similar to those described previously, but were suggestive of a residual cyst primarily due to the absence of teeth 24 and 25. An interesting observation in our literature review of the previous cases of GOC reported in India showed that 7 out of 19 cases were also located in edentulous or partially edentulous areas (where teeth were extracted previously). This could indicate that GOC may possibly grow within the jaws without any symptoms in the initial stages, and cause swelling and cortical expansion only at a later date.

**Table 1:** Clinical data of 19 cases of GOC previously reported in India along with the present case

Authors of the Case Report	Age of Patient (years)	Sex	Swelling	Pain	Location
Baliga et al (1997) [6]	26	Male	Present	Present	Left anterior maxilla 21-22
Jose et al (2000) [7]	18	Female	Present	Present	Right maxilla 16-17 and maxillary sinus
Krishnamurthy et al (2009) [5]	42	Female	Present	Absent	Anterior mandible, extending from right to left body of mandible 36-45
	21	Male	Present	Absent	Left mandible, from midline till ascending ramus
Prabhu et al (2010) [8]	47	Female	Present	NR	Right maxilla 14-18 (missing 16)
Rao et al (2010) [9]	60	Female	Present	Absent	Left maxilla 26-28 and maxillary sinus (missing 27, 28)
Guruprasad et al (2011) [10]	17	Female	Present	Present	Right posterior maxilla and maxillary sinus
Amberkar et al (2011) [11]	29	Male	Present	Present	Bilateral - right maxilla 14-16 and left maxilla 24-26
Purohit et al (2014) [2]	30	Female	Present	Absent	Right maxilla 15-17
Shah M et al (2014) [12]	40	Male	Present	Present	Anterior mandible – left to right side 36-43
Tambawala et al (2014) [13]	17	Female	Present	Present	Right mandible – angle and ramus
Mittal et al (2015) [14]	17	Female	Present	Present	Left anterior to right posterior mandible 34-47
Faisal et al (2015) [15]	11	Male	Present	Absent	Anterior mandible, extending from right to left body of mandible 36-45
Raju et al (2015) [16]	65	Female	Present	Absent	Anterior mandible, extending from right to left body of mandible (missing 31, 32, 41, 42)
Anchlia et al (2015) [17]	63	Female	Present	Present	Right anterior mandible, 31-45
Shah A et al (2016) [3]	25	Male	Present	Absent	Left mandible 35-37 (missing 36)
	30	Female	Present	Absent	Right mandible 46 (missing 46)
Surej Kumar et al (2016) [18]	46	Female	Present	Absent	Anterior maxilla 12-13
Chandra et al (2016) [19]	70	Female	Present	Present	Anterior maxilla 21-23
**Present Case**	**64**	**Female**	**Present**	**Present**	**Left maxilla 23-26**

**Table 2:** Radiographic data of 19 cases of GOC previously reported in India along with the present case

Authors of the Case Report	Radiographic Appearance	Margins / Borders	Internal Structure	Root Resorption	Tooth / Root Displacement	Cortical Expansion	Impacted Tooth
Baliga et al (1997) [6]	Unilocular	Well-defined, Corticated	Radiolucent	Absent	Present 22	Palatal	Absent
Jose et al (2000) [7]	NR	NR	Radiolucent	NR	NR	Buccal, palatal	Absent
Krishnamurthy et al (2009) [5]	Multilocular	Well-defined	Radiolucent	Edentulous	Edentulous	Buccal, lingual	Absent
	Multilocular	Well-defined, corticated	Radiolucent	Absent	Absent	Buccal	Absent
Prabhu et al (2010) [8]	Unilocular	Well-defined	Radiolucent	NR	NR	Buccal, palatal	Absent
Rao et al (2010) [9]	Unilocular	Well-defined	Radiolucent	Absent	Absent	Buccal	Absent
Guruprasad et al (2011) [10]	Unilocular	Well-defined	Radiolucent	Absent	Absent	NR	Present 18
Amberkar et al (2011) [11]	Unilocular	Well-defined	Radiolucent	Absent	Present 14, 15	NR	Absent
Purohit et al (2014) [2]	Unilocular	Well-defined	Radiolucent	Present 16	Present 15, 17	Buccal	Absent
Shah M et al (2014) [12]	Multilocular	Well-defined, Corticated	Radiolucent	Absent	Absent	Buccal	Absent
Tambawala et al (2014) [13]	Multilocular	Well-defined	Radiolucent	Present 47	Absent	Buccal, lingual with perforation	Absent
Mittal et al (2015) [14]	Multilocular	Well-defined	Radiolucent	NR	NR	NR	Absent
Faisal et al (2015) [15]	Multilocular	Well-defined, Corticated	Radiolucent	Absent	Absent	Buccal, lingual, with perforation	Absent
Raju et al (2015) [16]	Multilocular	Well-defined	Radiolucent	Absent	Absent	NR	Absent
Anchlia et al (2015) [17]	Unilocular	Well-defined, Corticated	Radiolucent	Absent	Present 43	NR	Absent
Shah A et al (2016) [3]	Unilocular	Well-defined, Corticated	Radiolucent	Absent	Absent	Buccal, lingual	Absent
	Unilocular	Well-defined, Corticated	Radiolucent	--	Absent	NR	Absent
Surej Kumar et al (2016) [18]	Unilocular	Well-defined, Corticated	Radiolucent	Absent	Present	Buccal	Absent
Chandra et al (2016) [19]	Multilocular	Ill-defined	Radiolucent	Edentulous	Edentulous	Buccal, palatal	Absent
**Present Case**	**Unilocular**	**Well-defined, Corticated**	**Radiolucent**	**Absent**	**Absent**	**Buccal**	**Absent**

NR: Not reported

Histologically, GOC shows certain characteristic features that were subdivided by Kaplan et al into major and minor criteria based on the frequency of occurrence of each feature in the previously reported cases [2]. At least focal presence of the major criteria is mandatory, while the presence of minor criteria further supports the correct diagnosis [3]. The major criteria include a lining of squamous epithelium of varying thickness which may show epithelial ‘spheres’ or ‘whorls’ with absence of basal palisading, cuboid-shaped eosinophilic (‘hobnail’) cells, and intraepithelial mucous / goblet cells or pools, with presence of glandular or duct-like structures [3, 13]. Papillary proliferation of the epithelial lining, multicystic appearance, and presence of ciliated or clear cells in the epithelium were included as minor criteria [13]. The present case showed most of the characteristic histopathological features of GOC described above. The histopathological features of GOC have been found to resemble a number of lesions having a wide clinico-pathologic spectrum ranging from other odontogenic cysts like lateral periodontal cyst to malignant neoplasms such as central low-grade mucoepidermoid carcinoma, and hence careful and detailed microscopic examination is essential in arriving at a correct diagnosis [5]. The exact treatment of choice of this lesion remains controversial, and varies from enucleation and curettage to local block excision depending on the size and aggressiveness [1]. Enucleation is preferred for small unilocular lesions, whereas management of large lesions includes enucleation with peripheral ostectomy for unilocular cases and marginal resection or partial jaw resection in multilocular cases [16]. Some of the previous cases reported in literature have been found to have an aggressive potential and hence a long-term follow-up is important to monitor for any signs of recurrence. The present case resembled a residual cyst and was thus treated by enucleation and curettage, and has been kept under regular observation till date, with no evidence of recurrence.

## Conclusion

GOC is an uncommon cystic lesion, with less than 200 cases reported world-wide till date. This cyst shows non-specific clinico-radiographic characteristics which may resemble a wide-spectrum of lesions. A final diagnosis can only be obtained after a careful histopathologic examination and a long-term follow-up is required to rule out recurrences.
